# Brain volumetry in fetuses that deliver very preterm: An MRI pilot study

**DOI:** 10.1016/j.nicl.2021.102650

**Published:** 2021-03-29

**Authors:** Lisa Story, Alice Davidson, Prachi Patkee, Bobbi Fleiss, Vanessa Kyriakopoulou, Kathleen Colford, Srividhya Sankaran, Paul Seed, Alice Jones, Jana Hutter, Andrew Shennan, Mary Rutherford

**Affiliations:** a Department of Women and Children’s Health, King’s College London, UK; b Centre for the Developing Brain, King’s College London, London, UK; c School of Health and Biomedical Sciences, RMIT University, Bundoora 3083, VIC, Australia; d Université de Paris, NeuroDiderot, Inserm, F-75019 Paris, France; e Centre for Medical Engineering, King’s College London, London, UK; f Fetal Medicine Unit, Guy’s and St Thomas’ Hospital London, UK; g Queen Mary University Medical School, UK

**Keywords:** CP, Cerebral Palsy, CPAP, Continuous Positive Airway Pressure, CSP, Cavum Septum Pellucidum, eCSF, extra Cerebro Spinal Fluid, IVH, Intra Ventricular Haemorrhage, MRI, Magnetic Resonance Imaging, PPROM, Preterm Premature Rupture of Membranes, PTB, Preterm Birth, SVR, Slice to Volume Reconstruction, TEA, Term Equivalent Age, Preterm birth, fetal MRI, Infection, Brain volume, Cortex, Cerebrospinal fluid

## Abstract

**Background:**

Infants born preterm are at increased risk of neurological complications resulting in significant morbidity and mortality. The exact mechanism and the impact of antenatal factors has not been fully elucidated, although antenatal infection/inflammation has been implicated in both the aetiology of preterm birth and subsequent neurological sequelae. It is therefore hypothesized that processes driving preterm birth are affecting brain development in utero. This study aims to compare MRI derived regional brain volumes in fetuses that deliver < 32 weeks with fetuses that subsequently deliver at term.

**Methods:**

Women at high risk of preterm birth, with gestation 19.4–32 weeks were recruited prospectively. A control group was obtained from existing study datasets. Fetal MRI was performed on a 1.5 T or 3 T MRI scanner: T2-weighted images were obtained of the fetal brain. 3D brain volumetric datsets were produced using slice to volume reconstruction and regional segmentations were produced using multi-atlas approaches for supratentorial brain tissue, lateral ventricles, cerebellum cerebral cortex and extra-cerebrospinal fluid (eCSF). Statistical comparison of control and high-risk for preterm delivery fetuses was performed by creating normal ranges for each parameter from the control datasets and then calculating gestation adjusted z scores. Groups were compared using *t*-tests.

**Results:**

Fetal image datasets from 24 pregnancies with delivery < 32 weeks and 87 control pregnancies that delivered > 37 weeks were included. Median gestation at MRI of the preterm group was 26.8 weeks (range 19.4–31.4) and control group 26.2 weeks (range 21.7–31.9). No difference was found in supra-tentorial brain volume, ventricular volume or cerebellar volume but the eCSF and cerebral cortex volumes were smaller in fetuses that delivered preterm (p < 0.001 in both cases).

**Conclusion:**

Fetuses that deliver preterm have a reduction in cortical and eCSF volumes. This is a novel finding and needs further investigation. If alterations in brain development are commencing antenatally in fetuses that subsequently deliver preterm, this may present a window for in utero therapy in the future.

## Highlights

• Fetuses that subsequently deliver very preterm have a reduction in cortical and extra cerebrospinal fluid volumes.
• If such alterations commence antenatally this suggests a role for earlier administration of neuroprotective agents.

## 1 Introduction

Very preterm birth (PTB), occurring<32 weeks gestation, accounts for 1.2% of all deliveries within England and Wales (National office of Statistics, 2019), every year. Although, survival of very preterm infants has increased over recent decades (Santhakumaran et al., 2018), this has been associated with an increase in long term morbidity (Saigal and Doyle, 2008). Neurodevelopmental sequelae account for a significant proportion of these complications: up to 7% of surviving infants will develop motor impairments in the form of cerebral palsy (CP) (Pascal et al., 2018) and up to 50% will suffer some form of cognitive, behavioural, attention or socialisation deficit (Montagna and Nosarti, 2016). The impact on individuals, their families and health care services is therefore considerable.

A reduction in regional brain volumes at term equivalent age (TEA) has previously been reported in children that were born preterm including white matter, deep gray matter and the brainstem, in addition to an increase in cerebrospinal fluid (CSF) (Padilla et al., 2015). Variable alterations in cortical development at term equivalent age (TEA), both in macro (Ball et al., 2012, Bouyssi-Kobar et al., 2018) and microstructure (Eaton-Rosen et al., 2017) using advanced MRI techniques have also been identified and assumptions to date have been that these are a consequence of the delivery process and the postnatal environment (Chau et al., 2013, Padilla et al., 2015). However, it remains unclear whether brain development in fetuses that deliver preterm is already altered prior to delivery.

Infection has been implicated in both the aetiology of PTB and the subsequent neurodevelopmental sequelae; approximately 80% of cases of PTB, have evidence of significant microbial colonization within placental parenchyma when delivery occurs before 28 weeks gestation (Onderdonk et al., 2008). Chorioamnionitis is also associated with brain pathology such as intraventricular haemorrhage (IVH) (Alexander et al., 1998, De Felice et al., 2001), white matter impairment (Anblagan et al., 2016) and is a risk factor for neurodevelopmental impairment at 10 years of age (Venkatesh et al., 2020). Pro-inflammatory cytokines within both maternal and fetal circulations have been linked to both overt and subtle white matter damage (Elovitz et al., 2006). Elevated levels of interleukin-8, a characteristic feature of intrauterine infection have additionally been shown to be associated with neurite density in preterm infants at TEA. Animal models have also implicated infection as the major aetiology for neuronal aberrations associated with spontaneous PTB (Burd et al., 2010), however, this has not been investigated prospectively in the human fetus.

We hypothesize that the processes driving preterm birth may also affect brain development in utero.

This study therefore aims to assess if brain development is altered in utero in fetuses who subsequently deliver at <32 weeks preterm using advanced MRI imaging and comparing with gestationally matched fetuses that subsequently deliver at term.

## 2 Materials and Methods

Women with pregnancies at high risk of preterm birth were prospectively recruited from St Thomas’ Hospital London between December 2015 and February 2020 (ethics 07/H0707/105, 19/SS/0032 and 16/LO/1573).

Inclusion criteria were: GA 18–32 weeks gestation and a high risk of preterm birth defined as either women with preterm premature rupture of membranes or asymptomatic women with either a history of previous PTB, late miscarriage > 16 weeks or cervical surgery with a > 50% risk of PTB < 32 weeks gestation based on an algorithm derived from quantitative cervico-vaginal fibronectin and cervical length (Kuhrt et al., 2015). Exclusion criteria included: twin pregnancies, fetuses with chromosomal or structural abnormalities, active labour, inability to give informed consent, BMI > 35, contraindications to MRI including recently sited metallic implant or claustrophobia.

A control cohort was obtained from existing datasets of uncomplicated pregnancies, who had volunteered to have a research MRI, from two other studies conducted within the department (the intelligent Fetal Imaging and Diagnosis project (www.iFINDproject.com) and the Placental Imaging Project (www.placentaimagingproject.org). Cases were selected based on a comparable gestational age range at the time of the MRI as the preterm cases, and where delivery occurred after 37 weeks gestation. All of these scans were undertaken on a 1.5 T or 3 T MRI scanner using the same protocols as described below and data was post processed using the same techniques.

### 2.1 Image acquisition

Following assessment of eligibility, women were invited to participate and written consent was obtained. A fetal MRI was performed on a Philips Ingenia (1.5 T); Philips Medical systems or a 3 T Philips Achieva system, (Best, the Netherlands) with a 28-channel (1.5 T) or 32-channel coil placed around the mother’s abdomen. The decision regarding which scanner to use was based on availability and individual study protocols. Women were successfully imaged without incident in a supine position (Hughes et al., 2016), with oxygen saturation and blood pressure monitoring throughout. The total scan length did not exceed one hour and an obstetrician was present throughout the duration of the scan, with a break offered midway through.

Imaging of the fetal brain was acquired using a T2-weighted single-shot turbo spin-echo (ssTSE) sequence acquired in three orthogonal planes using the following scanning parameters: TR = 25991 ms, TE = 80 ms, slice thickness of 2.5 mm, slice overlap of 1.25 mm and flip angle = 90°. Imaging of the entire uterus was also performed to include the whole fetal body in at least two planes and at least three acquisitions. The following scanning parameters were used: TR = 25991 ms, TE = 80 ms, slice thickness of 2.5 mm, slice overlap of 1.25 × 1.25 × 1.25 mm on 1.5 T, and 1.21 × 1.21 × 1.5 or 1.25 × 1.25 × 2.5 mm on 3 T, with a flip angle = 90°.

All scans were reviewed for image quality.

### 2.2 Data Post-processing

Snapshot MRI with Volume Reconstruction (SVR) was used to generate 3D reconstructed images, as previously described (Jiang et al., 2014, Kuklisova-Murgasova et al., 2012). Image registration was performed to align all images obtained based on the assumption of a rigid body, of constant shape and size, performing an unknown motion. Images were registered onto a self-consistent anatomical space of the fetal brain (volume with least motion), and using a scattered interpolation approach, all measured voxel intensities are used to reconstruct the 3D fetal brain with an accuracy of 0.3 mm. The reconstructed 3D volumetric data sets have high resolution, high signal-to-noise ratio, and full brain coverage essential for reliable volumetric analysis. The 3D fetal volumetric brain data were orientated into standard axial, coronal, and sagittal projections, and the voxel size was interpolated from a reconstruction voxel size of 1.18 × 1.18 × 1.18 mm to 0.2 × 0.2 × 1 mm to aid visual display, delineation of finer structures for volumetric segmentation, and to assist in the placement of anatomical markers.

**Fig. 1:**
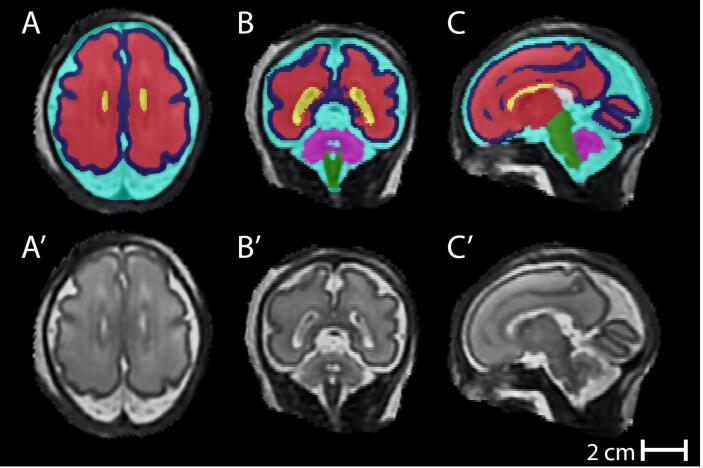
regional brain volumes in a control fetus at 28^+2^ weeks gestation acquired on a 3 T MRI system in the axial (A), coronal (B) and saggital planes (C) for supratentorial brain volume – red, lateral ventricles - yellow, extra-cerebral cerebrospinal fluid – turquoise, cerebellar volume - pink and cortical volume - blue. The original reconstructed datasets can be seen in A’, B’ and C’. (For interpretation of the references to colour in this figure legend, the reader is referred to the web version of this article.)

Reconstructed T2-weighted fetal images were segmented using a modified version of *The Developing Brain Region Annotation With Expectation-Maximization (Draw-EM*) MIRTK Package, an automated tissue segmentation algorithm, originally optimised for the neonatal brain (Makropoulos et al., 2016, Makropoulos et al., 2014, Makropoulos et al., 2018). Tissue segmentations were visually inspected for accuracy and mislabelled voxels were manually edited using ITK-SNAP(Yushkevich et al., 2006). Fine editing duration varied between one to three hours per brain based on the quality of initial reconstruction and the resultant intensity-based segmentation. An example of segmented brain regions can be seen in Fig. 1.

Images which were not successfully processed (n = 21), due to excessive fetal motion or insufficient T2 acquisitions, through the automated pipeline, were manually segmented. Comparison between automated and manual segmentation had been compared (intraclass correlation co-efficient ICC 0.97) and good intra- (ICC > 0.99) and inter- (ICC = 0.98) observer variability confirmed. All segementations were undertaken by three members of the research team (LS, AJ, AR).

As previously described (Kyriakopoulou et al., 2017), supratentorial brain tissue volume was defined as the brain tissue above the tentorium, i.e., excluding the brainstem, cerebellum, and all cerebrospinal fluid (CSF) spaces including the ventricles. Total ventricular volume was defined as the volume of both left and right lateral ventricles including the choroid plexus but excluding the third and fourth ventricles and cavum septum pellucidum and vergae (CSP). Cortical volume represented the total cerebral cortical gray matter. The total cerebellar volume measurement included both the cerebellar hemispheres and the vermis volumes and excluded the fourth ventricle. Extra-cerebral CSF included all intracranial CSF spaces surrounding the supratentorial brain tissue, those infratentorially surrounding the brainstem and cerebellum and included the interhemispheric fissure space but not any ventricular structure or the CSP.

Details of maternal demographics, timing of steroid administration in the preterm cohort, delivery and neonatal parameters were collected including: gestation at delivery, sex of infant, birthweight, birthweight centile, neonatal unit admission, number of days of intensive and high dependency care, any brain abnormalities detected on postnatal ultrasound or MRI imaging, invasive ventilation, Continuous Positive Airway Pressure (CPAP), the need for supplemental oxygen, sepsis and necrotizing enterocolitis. Placental histology was reviewed where available for evidence of chorioamnionitis.

### 2.3 Statistical analysis

Data were assessed for normality using distributional plots of standardized residuals. Demographic and neonatal outcome data were analysed using a student *t*-test where data were continuous and Chi-squared where categorical. For the control cases the Wright and Royston xrimel method was used to estimate normal growth trajectory of each brain region between 20 and 32 weeks gestation (Royston and Wright, 1998). Z scores were then created for each observation, accounting for the effects of gestation. ROC curves were generated for low regional brain volumes as predictors of PTB.

Student t tests were then use to compare a scores between the control and preterm groups. This analysis was repeated to assess if magnet strength affected volume measurements. To additionally ensure compatibility between volumetric data acquired from 1.5 T and 3 T reconstructions, a healthy pregnant volunteer had previously been scanned on both scanners on the same day. Volumetric data were compared and no significant differences were found between the images across any of the key fetal brain segmentation regions; volumes were within the expected ±5% threshold. Statistical analysis was performed using the SPSS software package (version 26, SPSS IBM) and STATA (version 16).

## 3 Results

Fifty-two women, at high risk of preterm birth, who satisfied the study entry criteria were approached during the study period: five declined to participate and 18 delivered prior to the MRI scan being undertaken. Twenty-nine MRIs were performed. Five women delivered after 32 weeks gestation, and hence the data was excluded.

Twenty four women therefore delivered < 32 weeks gestation: 18 had ruptured membranes and six had intact membranes at the time of the MRI scan. In the fetuses with ruptured membranes the median number of days from rupture to MRI was five (range 1–20). Eleven women were scanned on a 1.5 T system and 13 on a 3 T system due to scanner availability and study protocols. The median number of days from MRI to delivery was 10 (range 0–48). Placental histology was undertaken in 20 of the 24 cases. Chorioamnionitis was present in 90% of these. None of the 24 women had features of growth restriction on antenatal ultrasound imaging (estimated fetal weight < 10th centile or abnormal umbilical artery Doppler velocimetry).

Eight-seven controls were identified from existing datasets: 42 were undertaken on a 1.5 T MR system and 45 on a 3 T. Ultrasound data at the time of MRI imaging or after was available for 57 of the controls (67%). None of these cases had evidence of growth restriction (estimated fetal weight < 10th centile or abnormal umbilical artery Doppler velocimetry). Thirty had no recorded growth scans after 20 weeks. Of these cases only 2 measured < 10th centile at delivery and none measured < 3rd centile.

Placental histology was available for 28 of the control cases, 15 of which had chorioamnionitis. It should however be noted that unless part of a study protocol placentas are not routinely sent at delivery for histological assessment. Also the development of chorioamnionitis in these cases was likely to have ensued during or just prior to labour onset.

Clinical characteristics of both the control cohort and pregnancies that delivered < 32 weeks gestation can be seen in Table 1.

**Table 1:** Clinical Characteristics of the Cohort.

Characteristic	Preterm cohort (n = 24)	Term cohort (n = 87)	P
Maternal age, y			
Mean (SD)	35.2 (4.8)	34(4)	0.15
Range	28–45	25–45	
Ethnicity (%)			
White	50	76	**0.002**
Black	25	16	
South Asian	20.8	1	
Other	4.2	6	
Smoking Status (%)			
Smoker	4.2	1.1	0.39
Ex-smoker	12.5	8	
Non-smoker	83.3	83.9	
Data unavailable	0	6.9	
GA at MRI, wk			
Mean (SD)	26.8 (3.2)	26.2 (2.3)	
Range	19.4–31.4	21.7–31.9	
GA at birth, wk			
Mean (SD)	28.6 (3.0)	40.1 (1.2)	
Range	20.1–31.9	37.1–42.1	
Birthweight, g	N = 22*		
Mean (SD)	1293 (320.3)	3449 (468.5)	
Range	605–1875	2485–4560	
Birthweight centile (%)	0	2.3	0.70
0–3	9.1	8.0	
3–10	13.6	24.1	
10–25	27.3	18.4	
25–50	40.9	27.6	
50–75	9.1	13.8	
75–90	0	3.4	
90–97	0	2.3	
97–100			
Sex of Infant n (%)			
Female	50	48.3	0.78
Male	45.8	51.7	
Undetermined	4.2	0	
Outcome n (%)			
Live to discharge Neonatal/intrapartum death	87.5	100	
	12.5	0	

*t*-test used for analysis where data was continuous and Chi-squared where data categorical

* for two of the intrapartum deaths at pre-viable gestations birthweight was not available from clinical records.

In the term cohort, no overt clinical pathology was noted on MRI in any of the fetal brains antenatally. However, of the preterm cohort one case imaged at 25^+2^ weeks gestation had bilateral small pseudocysts present, one fetus imaged at 30^+4^ weeks gestation was noted to have a prominent cisterna magna, one fetus at 29^+6^ had a prominent venous sinus and another fetus at 26^+2^ was noted to have markedly reduced eCSF on the clinical report.

**Fig. 2:**
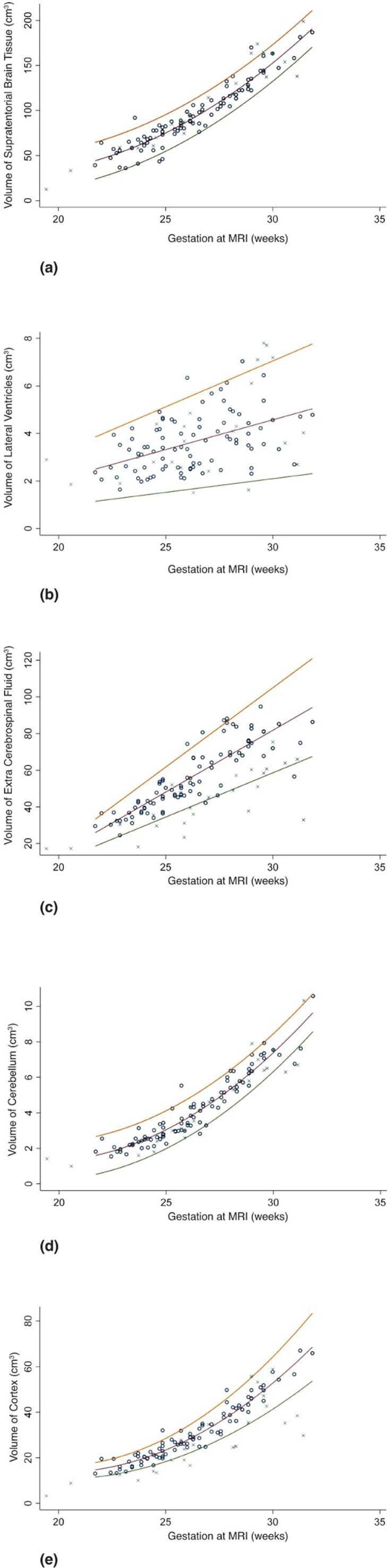
regional brain volumes for (a), supratetorial brain volume (b), lateral ventricles (c), extra-cerebral cerebrospinal fluid (d) cerebellar volume (e) cortical volume in fetuses that deliver<32 weeks gestation (crosses) and those that deliver at term (circles). Normal ranges and 3 50th and 97th centiles for are illustrated derived from the control poulation. M = median, S = standard deviation Z = Z score.

Normal ranges, with 3rd, 50th and 97th centiles indicated, for each regional brain volume can be seen in Fig. 2. Correlations between brain volumes and gestation of scan were extremely high for cortex (r^2^ = 0.902), supratentorial brain tissue (r^2^ = 0.884) and cerebellum (r^2^ = 0.881) but lower for the eCSF (r^2^ = 0.736). and lateral ventricles (r^2^ = 0.189). Fig. 2 also shows regional brain volumes in fetuses that delivered < 32 weeks gestation. There were no significant differences in supratentorial brain tissue, cerebellar or ventricular volume, however there was a reduction in eCSF and cortical volume in those fetuses who subsequently delivered preterm compared to controls (See Table 2). These results were not affected by the MRI system used (See Table 3). eCSF:supratentorial brain tissue volumes was also reduced in the preterm cohort (p < 0.001), however, cortex:supratentorial brain tissue was not. Numbers were too small to compare fetuses with and without chorioamnionitis.

**Table 2:** Z scores of antenatal regional brain volumes in fetuses that < 32 weeks gestation in comparison with the normal range derived from fetuses that subsequently delivered at term.

Variable	Mean and SD Z score preterm group	95% confidence interval of the difference	P value
Supratentorial brain volume (cm3)	0.25 (1.422)	−0.278 – 0.800	0.338
Ventricular volume (cm3)	0.083 (1.442)	−0.596–0.693	0.878
Extracerebral cerebrospinal fluid volume (cm3)	−1.667 (1.632)	−2.374- −0.936	**<0.001**
Cerebellar volume (cm3)	−0.542 (1.474)	−01.211–0.105	0.096
Cortical volume (cm3)	−2.042 (2.074)	−2.646- −1.414	**<0.001**

**Table 3:** Comparison of Z scores on 1.5 and 3 Tesla MRI systems for antenatal regional brain volumes in fetuses that delivered < 32 weeks gestation and those that subsequently delivered at term.

Variable	Mean and SD Z score control group 3 T scanner	Mean and SD Z score control group 1.5 T scanner	Mean and 95% CI of the difference	P value	Mean and SD Z score preterm group 3 T scanner	Mean and SD Z score preterm group 1.5 T scanner	Mean and 95% CI of the difference	P value
Supratentorial brain volume (cm3)	0.044 (1.021)	0.273 (1.421)	0.068 (−0.406–0.542)	0.777	0.231 (1.481)	0.273 (1.421)	0.068 (−0.406 – 0.545)	0.944
Ventricular volume (cm3)	0.067 (1.053)	0.000 (1.082)	−0.067 (−0.522–0.389)	0.772	0.539 (1.330)	−0.455 (1.440)	0.570 (−2.166–0.180)	0.096
Extracerebral cerebrospinal fluid volume (cm3)	0.111 (1.027)	−0.143 (1.026)	−0.254 (0.220 - −0.692)	0.252	−1.615 (1.981)	−1.727 (1.91)	0.656 (−1.481–1.257)	0.866
Cerebellar volume (cm3)	−0.1556 (1.167)	0.191 (0.943)	0.346 (−0.105–0.797)	0.131	−0.308 (1.377)	−0.818 (1.601)	−0.510 (0.616- −1.795)	0.417
Cortical volume (cm3)	0.111 (0.982)	−0.143 (1.160)	−0.254 (0.230- −0.714)	0.275	−1.549 (2.537)	−2.636 1.206)	−1.098 (0.792- −2.764)	0.203

A post hoc partial correlation analysis was performed revealing a positive correlation between eCSF and cortical volume, accounting for the effects of gestation in the control group (p = 0.007 r = 0.305) but not the preterm group (p = 0.26 r = 0.275). Numbers were also limited with regards to fetuses that had ruptured membranes and those that did not, however findings persisted when the groups were analysed separately.

The expected mean supratentorial volume (cm^3^) at a given gestational age (GA) was given using the following model:

M = (gestation at MRI – 26.324) + ((gestation at MRI)^2^−6.930) + 91.937

S = 0.023567

Z = (Volume of supratentorial brain tissue - M)/S

The expected mean ventricular volume (cm^3^) at a given gestational age (GA) was given using the following model:

M = (gestation at MRI – 26.324) + 3.654

S = 0.288 × M

Z = (Volume of ventricles - M)/S

The expected mean extra cerebrospinal volume (cm^3^) at a given gestational age (GA) was given using the following model:

M = 56.997 + 6.741 × (gestation of MRI - 26.32419348)

S = 0.1509086 × M

Z = (Volume of eCSF- M)/S

The expected mean cerebellar volume (cm^3^) at a given gestational age (GA) was given using the following model:

M = (gestation at MRI –2.632) +(gestation at MRI)^2^ – 6.930) + 3.952

S = 0.567

Z = (Volume of cerebellum - M)/S

The expected mean cortical volume (cm^3^) at a given gestational age (GA) was given using the following model:

M = 29.262 + -155.411 × (gestation at MRI/10 − 2.632419348 + 38.92872 × ((gestation of MRI/10) ^2^ –6.930

S = 0.115 × M

Z = (Volume of cortex- M)/S

Twenty-one of the 24 women (all those who were of viable gestations) born preterm, received steroids prior to the MRI scan. Only one had received magnesium sulphate prior to the MRI scan (this baby died at 24^+3^ weeks gestation immediately after delivery). Two other babies died in the intra-partum period. One was imaged at 19^+4^ weeks and delivered at 20^+1^ and the other imaged at 20^+4^ and delivered at 22^+0^ weeks gestation: no remarkable features were noted on antenatal MRI.

Delivery details can be seen in Table 1 and short-term outcome parameters seen in Table 4. No correlation was found between regional brain volumes and neonates with abnormal brain imaging (ultrasound or MRI n = 10) compared to neonates without abnormal brain imaging (See Table 4).

**Table 4:** Short-term outcomes of fetuses born < 32 weeks gestation. None of the control group experienced any neonatal complications.

	Number of cases of surviving infants (n = 21)
Days of Care	
ITU	
*Median (range)*	3
*Range*	0–30
HDU	
*Median*	9
*Range*	1–48

Neurological Outcomes	
Intra-ventricular haemorrhage on US	8
*Grade 1*	3
*Grade 2*	4
*Grade 3 (requiring shunt)*	1
Cerebral infarction	1

Respiratory Outcomes	
Days of intubation	
*Median*	1
*Range*	0–24
Days of CPAP	
*Median*	2
*Range*	0–16
Bronchopulmonary dysplasia	2

Gastrointestinal Complications	
Necrotising Enterocolitis	1

**Fig. 3:**
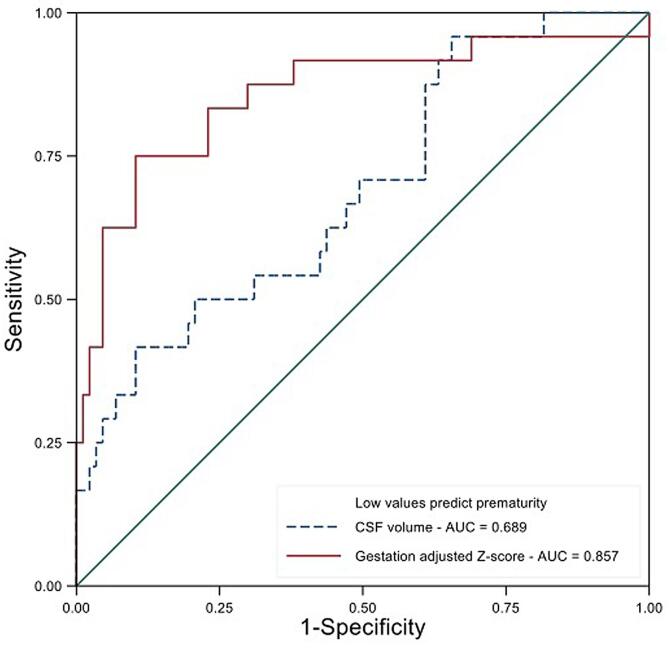
ROC curve of prediction of prematurity from antenatal MRI scans for gestation adjusted z scores of extracereberospinal fluid volume and cortical volume.

The gestation adjusted regional brain volumes were tested as predictors of PTB. Low eCSF volume and low cortical volume z scores were the best predictors (see Fig. 3).

Six of the fetuses that delivered preterm had postnatal MRI. Three of these were research scans performed as part of the developing Human Connectome Project. Three were performed due to abnormalities identified on clinically performed ultrasound examinations. Of the three clinical scans, one showed widespread cystic encephalomalacia, another had grade 2 IVH and the third had grade 3 IVH with severe post haemorrhagic ventricular dilation. Of the research MRIs, one was normal, another had punctate lesions in the periventricular white matter and the third had a small cortical infarction. Details of these scans can be seen in Table 5.

**Table 5:** Details of postnatal MRI where available from the preterm cohort.

Case	Gestation at antenatal MRI	Antenatal MRI Report	Gestation at Delivery	Gestation at Neonatal MRI (postmenstrual age)	Findings of Neonatal MRI	Gestation at second neonatal MRI (postmenstrual age)	Findings of second neonatal MRI
1	25 + 2	Normal	28 + 4	35 + 6	Punctate WM lesions mild vermis rotation	38 + 4	Persistent punctate lesions mild vermis rotation
2	29 + 2	Lateral ventricles 9.3 and 7.5 mm	31 + 4	34 + 3	Small left cortical infarct	44 + 4	Previous infarction involuted
3	30 + 4	Prominent posterior fossa	31 + 0	33 + 4	normal	40 + 6	No focal pathology
4	26 + 1	Normal	28 + 1	36 + 1	widespread cystic encephalomalacia,	Not performed	
5	26 + 2	Reduced CSF no evidence of brain pathology	28 + 1	35 + 5	IVH	Not performed	
6	30 + 0	Normal	30 + 2	32 + 3	Bilateral IVH with severe ventricular dilation. Punctate WM lesions	Not performed	

WM = white matter. IVH = intraventricular haemorrhage.

## 4 Discussion

This study has demonstrated that fetuses that deliver very preterm had a significant reduction in cortical and extra-cerebral spinal fluid volumes antenatally compared with fetuses that subsequently delivered at term. A low extra cerebrospinal fluid volume and low cortical volume z scores appeared to be a good predictors of preterm birth. There was no difference in regional volumes for the cerebellum, lateral ventricles or supra-tentorial brain tissue. There were no signs of overt acquired focal injury on antenatal MRI. In addition, ten of the fetuses delivered preterm had evidence of acquired injury on postnatal imaging (MRI or ultrasound).

Preterm infants have a high risk of both haemorrhagic, e.g. germinal matrix/intraventricular injury and of white matter injury, e.g punctate white lesions, and less commonly venous infarction or cystic periventricular leukomalacia (Hinojosa-Rodriguez et al., 2017). These lesions may be evident on US or MRI soon after delivery although it is unclear whether the injurious process may start prior to labour onset. Thomason et al used functional MRI to assess fetuses that subsequently delivered preterm finding a reduction in neural connectivity in a left-hemisphere pre-language region (Thomason et al., 2017). Although, only 14 fetuses that delivered preterm and 18 that delivered at term were included in their study and the gestation at delivery was heterogeneous, ranging from 24 to 35 weeks gestation thereby also encompassing late preterm birth, results support our finding that alterations in brain development may commence antenatally in fetuses that subsequently deliver preterm.

The majority of studies assessing development of the preterm neonatal brain acquire imaging at TEA. This provides some ability to predict outcome in the presence of acquired brain lesions (Plaisier et al., 2014) but also allows a comparison of brain appearances and maturation with term born controls. Alterations in regional brain volumes, in the absence of overt focal injuries, have been reported at TEA in infants born preterm include: a reduction in cortical gray matter, white matter, deep gray matter and in the brain stem volume, and an increase in CSF (Padilla et al., 2015). Reductions in both white and grey matter volumes (Back, 2017, Peterson et al., 2000), cortical volumes (Inder et al., 2005) and cortical surface area (Kapellou et al., 2006, Rathbone et al., 2011) at TEA have been correlated with later adverse neurodevelopmental outcomes. It has previously been proposed that abnormalities on MRI scans in infants born preterm were attributable to disturbances of developmental processes during the neonatal period or delivery (Chau et al., 2013, Padilla et al., 2015, Ball et al., 2012, Ball et al., 2010, Anjari et al., 2009). However, the aetiology of the brain pathology associated with preterm birth is likely to be complex and multifactorial, also encompassing the antenatal period.

A reduction in both eCSF volume and cortical volume prior to delivery has been found in this study. Extra cerebral CSF has historically been considered to provide mechanical protection of the brain, however in recent years its pivotal role in brain development has been recognized. It has roles in the transportation of growth factors which regulate progenitor cell production (Lehtinen et al., 2011) and during cortical development(Mashayekhi et al., 2002, Miyan et al., 2003, Spector et al., 2015). Our findings of both a reduction in eCSF and cortical volume may be indicative of a disturbance in this relationship.

Our observed reduction in eCSF volume is in contrast to the findings in preterm born neonates where the eCSF volume appears increased when imaged at term equivalent age (Inder et al., 2005). Such increases may either reflect tissue atrophy or suboptimal growth or an alteration in the balance between CSF production and absorption. So called “benign” enlargement of the subarachnoid space may also occur postnatally in ex -preterm infants usually associated with an increase in head circumference centile (Kuruvilla, 2014). However, a previous MRI study in preterm infants, shortly after birth, showed that sepsis was associated with a reduction in CSF, volumes (although this was likely to also have included both extra-cerebral and ventricular CSF) and with subsequent reduced intracranial growth rates (Gui et al., 2019).

CSF plays a role in the removal of inflammatory cytokines and proteins secreted by neurons which may otherwise accumulate in brain tissue and have pathological effect on brain development (Johanson et al., 2008, Xie et al., 2013). CSF protein content has additionally been found to be altered in preterm born infants (Boardman et al., 2018), undergoing lumbar puncture for suspected sepsis compared with term born infants with suspected sepsis and overall these changes represented increased prototypical pro-inflammatory responses. Although brain levels of these pro-inflammatory compounds could not be measured in their study, the levels of these significantly altered molecules were assessed in microglial cells isolated from a mouse model of inflammatory brain injury of the preterm born infant (Van Steenwinckel et al., 2019). It was noted that, many of the same molecules altered in the CSF of the human infants, were also altered in the mouse model, revealing a temporally regulated pro-inflammatory response in the brain’s immune cells that would also signal an injurious environment. Within this study 90% of preterm placentas analysed had evidence of chorioamnionitis, which is in keeping a link between exposure to inflammation and reduced CSF volume. It is possible that a reduction in CSF volume may alter concentrations of inflammatory cytokines and growth factors which may have later implications for brain development. The fact that alterations in regional brain volumes, other than the cortex, were not observed in this study may simply be a function of the fact that during the antenatal period these processes have only just commenced.

Alterations in cortical maturation and microstructure have been previously identified in preterm delivered infants when imaged at TEA. In addition to a reduction in cortical volume (Padilla et al., 2015), diffusion tensor imaging demonstrated higher diffusivity in the prefrontal, parietal, motor, somatosensory and visual cortices in preterm infants at TEA compared to term born controls (Bouyssi-Kobar et al., 2018). This may represent reduced dendritic and synaptic density. Our finding of reduced cortical volumes in the fetus suggests that the cortical substrate may already be altered by the time of delivery in addition, to cortical development being influenced by postnatal factors. The underlying biology for these antenatal diminished volumes remains unclear.

Within our study 90% (19/20) of placentas analysed had evidence of chorioamnionitis, which would support a link between exposure to inflammation and reduced CSF volume. In a mouse model of preterm birth, where intrauterine inflammation was mimicked (by intrauterine infusion of lipopolysaccharide,) an increase in cytokine mRNA in whole fetal brain was found in addition to disrupted fetal neuronal morphology (Burd et al., 2010). Similar findings of neuroinflammation and altered cortical development are reported by others, including when employing a prolonged exposure to inflammation across the window of development in the mouse (Favrais et al., 2011, Fleiss et al., 2015, Stolp et al., 2019) and the sheep (Gussenhoven et al., 2018) equivalent to 22–32 weeks of human gestation.

## 5 Limitations

Although numbers at present are small and include fetuses both with and without ruptured membranes at the time of imaging this dataset is novel. Imaging fetuses at high risk of preterm delivery prospectively prior to birth is challenging and few centres in the world are equipped to undertake such a study as it requires both acurate identification of high risk pregnancies and rapid access to MRI scanners to perform imaging prior to delivery.

It should be noted that there is disparity in ethnicity between women in the control and group and those that delivered preterm. Marginal associations have previously been reported between regional brain volumes and ethnicity in the infant brain(Knickmeyer et al., 2017) however, this has not previously been explored in the fetus. In addition we did not have socioeconomic information regarding the study participants which has association with both preterm birth and brain injury(Benavente-Fernandez et al., 2020).

The interval between antenatal MRI scan and delivery was variable and in cases of membrane rupture, the time between rupture and imaging was also variable. However, correctly identifying and undertaking an MRI scan in women at high risk of delivery very preterm was a strength of this study. Participants either had to have ruptured membranes or a reduction in cervical length and increased levels of quantitative fetal fibronectin, indicating that the pathological processes culminating in preterm birth had already begun prior to the time of antenatal imaging.

## 6 Future work

These findings need to be validated in a larger group of fetuses, in an ethnically diverse sample, that subsequently deliver preterm. More advanced MR techniques will improve characterization of antenatal brain development in both control fetuses and those at high risk of preterm birth. Recent optimization of antenatal diffusion imaging (Lockwood Estrin et al., 2019) will give insight into tissue microstructure, such as alterations in cellular density and synaptic development which may preceed alterations in regional brain volumes. Specific areas within supratentorial brain tissue can also be evaluated such as the basal ganglia and thalami. In addition, histological studies to define biological substrates of cortical microstructure in preterm infants with and without signs of chorioamnionitis could substantiate in vivo imaging findings. Long term follow up of participants with postnatal imaging will help to establish the neurodevelopmental significance of these early brain findings and any associated fetal inflammatory responses. It would also help elucidate the relationship to acquired peripartum brain injuries, accounting for multiple confounding factors in the perinatal period. Further research regarding factors governing eCSF volume and its effect on ongoing brain development are required.

If pathological processes altering brain development are commencing antenatally this supports efforts for timely antenatal administration of neuroprotective agents. Although Magnesium Sulphate is currently recommended in pregnancies in preterm labour(NICE, 2015) this is usually given in labour itself. Future strategies may be better deployed at targeting the antenatal point at which pregnancies are deemed to be at high risk of preterm delivery, for example at the time of membrane rupture or when cervical shortening and a rise in quantitative fetal fibronectin occurs in order to modify very early laterations in brain development.

## Author contributions

LS and MR devised the study. LS recruited study participants. KC undertook the MRI scans. JH optimised data acquisition. AD PP and VK performed data post processing. LS, AD and AJ performed fine segmentation editing. LS and PS performed the statistical analysis. Review and editing was undertaken by LS, BF, SS, AS and MR.

## Declaration of Competing Interest

The authors declare the following financial interests/personal relationships which may be considered as potential competing interests: A.H.S is the chief investigator on a number of trials funded by NIHR and charity sources related to preterm birth prediction and prevention. Hologic Biomedical and Qiagen have provided samples for these studies. Hologic have provided funding (paid to the institution) to evaluate technical performance of their samples. Clinical Innovations provided the membrane rupture test used in this study. No other conflicts of interest.
